# Evolution of *CCL16* in Glires (Rodentia and Lagomorpha) shows an unusual random pseudogenization pattern

**DOI:** 10.1186/s12862-019-1390-7

**Published:** 2019-02-20

**Authors:** Fabiana Neves, Joana Abrantes, Ana M. Lopes, Luciana A. Fusinatto, Maria J. Magalhães, Wessel van der Loo, Pedro J. Esteves

**Affiliations:** 1 CIBIO, InBIO - Research Network in Biodiversity and Evolutionary Biology, Universidade do Porto, Campus de Vairão, Rua Padre Armando Quintas, 4485-661 Vairão, Portugal; 2 UMIB/UP – Unidade Multidisciplinar de Investigação Biomédica/Universidade do Porto, Porto, Portugal; 3 Departamento de Ecologia, Instituto de Biologia Roberto Alcântara Gomes, Universidade do Estado do Rio de Janeiro (UERJ), R. São Francisco Xavier 524, Rio de Janeiro, RJ CEP 20550-013 Brazil; 4 Departamento de Biologia, Faculdade de Ciências da Universidade do Porto, Rua do Campo Alegre, s/n, 4169-007 Porto, Portugal; 5 CITS - Centro de Investigação em Tecnologias de Saúde, CESPU, Gandra, Portugal

**Keywords:** Chemokine ligands, *CCL16*, Evolution, Glires, Pseudogenization

## Abstract

**Background:**

The C-C motif chemokine ligand 16 (CCL16) is a potent pro-inflammatory chemokine and a chemoattractant for monocytes and lymphocytes. In normal plasma, it is present at high concentrations and elicits its effects on cells by interacting with cell surface chemokine receptors. In the European rabbit and in rodents such as mouse, rat and guinea pig, *CCL16* was identified as a pseudogene, while in the thirteen-lined ground squirrel it appears to be potentially functional. To gain insight into the evolution of this gene in the superorder Glires (rodents and lagomorphs), we amplified the *CCL16* gene from eleven Leporidae and seven Ochotonidae species.

**Results:**

We compared our sequences with *CCL16* sequences of twelve rodent species retrieved from public databases. The data show that for all leporid species studied *CCL16* is a pseudogene. This is primarily due to mutations at the canonical Cys Cys motif, creating either premature stop codons, or disrupting amino acid replacements. In the Mexican cottontail, *CCL16* is pseudogenized due to a frameshift deletion. Additionally, in the exon 1 (signal peptide), there are frameshift deletions present in all leporids studied. In contrast, in *Ochotona* species, *CCL16* is potentially functional, except for an allele in Hoffmann’s pika. In rodents, *CCL16* is functional in a number of species, but patterns of pseudogenization similar to those observed in lagomorphs also exist.

**Conclusions:**

Our results suggest that while functional in the Glires ancestor, *CCL16* underwent pseudogenization in some species. This process occurred stochastically or in specific lineages at different moments in the evolution of Glires. These observations suggest that the *CCL16* had different evolutionary constrains in the Glires group that could be associated with the CCL16 biological function.

**Electronic supplementary material:**

The online version of this article (10.1186/s12862-019-1390-7) contains supplementary material, which is available to authorized users.

## Background

Chemokines are small pleiotropic proteins of low-molecular weight with important roles in inflammation, homeostasis and immune response [1, 2]. Chemokines are only found in vertebrates and are classified according to their conserved N-terminal cysteine residues into CC, CXC, XC, CX3C and CX (only identified in zebrafish) [3, 4]. In CC chemokines, both N-terminus cysteines are juxtaposed. Chemokines are able to exert their function through the interaction between the residues located in both the extracellular loops and the NH_2_-terminus and the chemokine receptors [5–7].

C-C motif chemokine ligand 16 (CCL16), also known as liver-expressed chemokine (LEC) or human CC chemokine (HCC)-4, is located in the macrophage inflammatory protein (MIP) region of the CC cluster. CCL16 is a strong pro-inflammatory chemokine and a chemoattractant for monocytes and lymphocytes, enhancing their adhesive properties [8, 9]. Commonly present at high concentrations in normal plasma, CCL16 elicits its effects on cells by interacting with cell surface chemokine receptors such as CCR1, CCR2, CCR5 and CCR8 [2]. In some mammalian species (human, mouse, rat, pig, cat, lion, European rabbit and domestic horse), CCR5 evolved under gene conversion with CCR2 [10–14]. In some, but not all leporid genera, the second external loop of CCR5 was altered by gene conversion with CCR2 [14, 15]. Indeed, the leporids European rabbit (*Oryctolagus cuniculus*), Amami rabbit (*Pentalagus furnessi*) and riverine rabbit (*Bunolagus monticularis*) CCR5 underwent gene conversion with CCR2, while cottontail rabbits (*Sylvilagus* spp.) and hares (*Lepus* spp.) have a normal CCR5. Since the second external loop is the target of chemokines, leporids are a good model to study the co-evolution of the chemokine receptors and their ligands [16]. In order to determine the consequences of this CCR5-CCR2 gene conversion, the CCR5 chemokine ligands have been studied in leporids. The study of *CCL8*, a prime ligand of CCR5, revealed that this gene was pseudogenized only in those species that underwent the CCR5 alteration, while it remained intact in hares and Eastern cottontail (*S. floridanus*) [16, 17]. In contrast, *CCL3, CCL4, CCL5* and *CCL11* genes were found to be functional in all studied leporids [18, 19]. While in rabbit, mouse and rat *CCL3* and *CCL4* are encoded by a single functional gene, they are duplicated in other rodents such as squirrel and guinea pig, being either functional or inactivated [20, 21]. *CCL14*, which is more closely related to *CCL16*, is functional in the Leporidae family while in Ochotonidae some species present a disrupted gene [22]. Interestingly, mouse and rat lack the *CCL14* gene [20]. Regarding *CCL16*, this gene is described to be pseudogenized due to different events in rabbit, mouse, rat and guinea pig [20, 23, 24].

The superorder Glires includes two orders, Rodentia and Lagomorpha, which diverged at approximately 82 million years ago (mya) [25]. Rodentia is the most diverse among placental mammals with 2277 species within 33 families [26]. Several phylogenies are proposed for rodents. According to Blanga-Kanfi et al. (2009), there are three main clades of rodents, the mouse-related clade, the squirrel-related clade and Ctenohystrica, that diverged ~ 73 mya. Lagomorpha includes two families, Ochotonidae (pikas) and Leporidae (rabbits and hares), that split at ~ 35 mya [27]. The Ochotonidae family is composed of only one genus, *Ochotona*, which is divided into four subgenera, *Pika*, *Ochotona*, *Conothoa* and *Lagotona* [28]. The Leporidae family comprises 11 genera *Poelagus*, *Pronolagus*, *Nesolagus*, *Oryctolagus*, *Caprolagus*, *Bunolagus*, *Pentalagus*, *Brachylagus*, *Sylvilagus*, *Lepus* and *Romerolagus*.

To elucidate the evolution of *CCL16* in the superorder Glires (rodents and lagomorphs), we sequenced the *CCL16* gene in 11 Leporidae and seven Ochotonidae species. We compared the sequences obtained with the *CCL16* sequences of 12 rodent species. Our results suggest that while functional in the Glires ancestor, *CCL16* underwent pseudogenization stochastically or in specific lineages at different moments in the evolution of Glires.

## Results

**Fig. 1:**
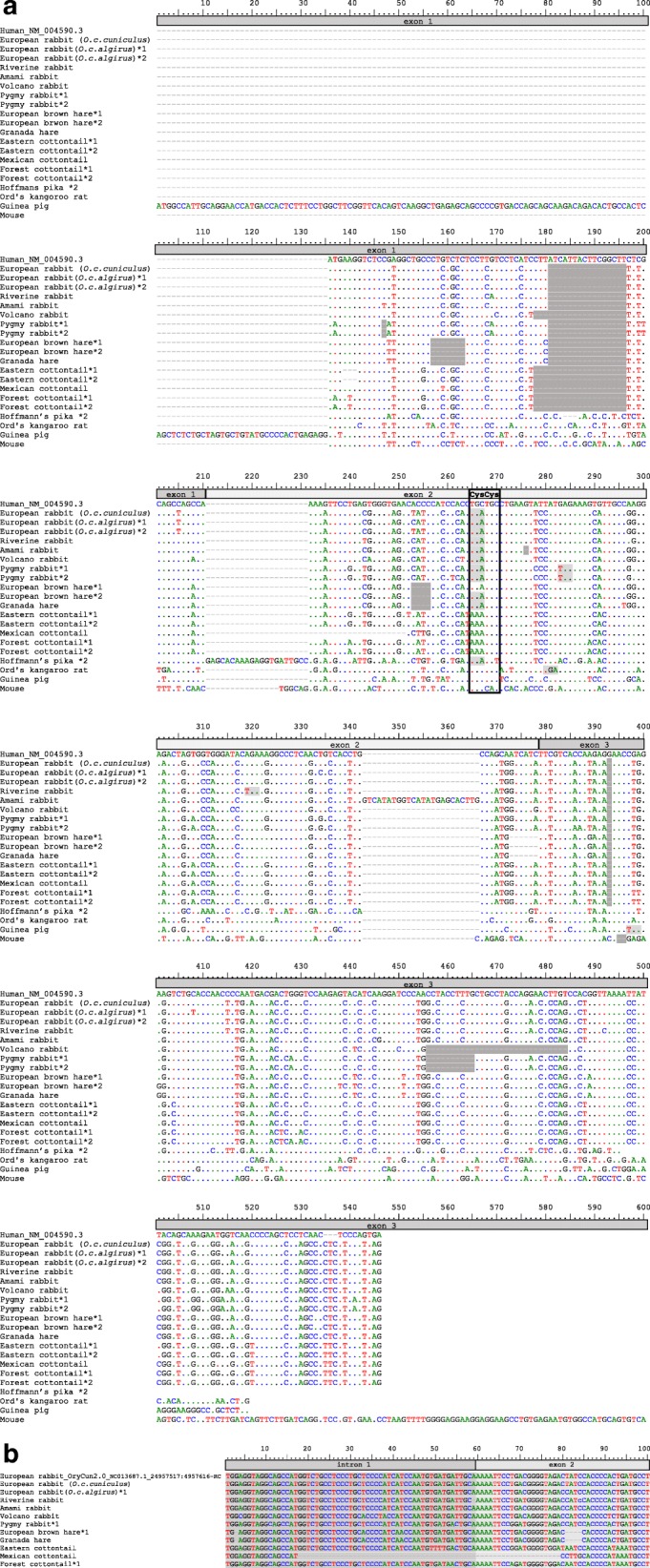
Detail of the nucleotide alignment for the different CCL16 pseudogenes (only a part of the mouse sequence is shown). *1 and *2 represent different alleles. The characteristic Cys Cys motif is boxed and the premature Stop codons are shaded in light grey. The frameshift mutations are shaded in dark grey (**a**). Detail of the Mexican cottontail deletion at the beginning of exon 2 (**b**)

In this study, we genetically characterized *CCL16* in lagomorphs. We further included the sequences available for several rodent species aiming at determine the evolution of this gene in the superorder Glires. The European rabbit sequence available in public databases (XM_08271780.1) was predicted by computational analyses and exon 1 was quite different from the remaining mammals (primates, artiodactyls or American pika). Thus, we amplified the exon 1 for leporids using the exon 1 of these mammals for primer design (Additional file 1 and Table 1). In most leporids, *CCL16* is a pseudogene due to a non-synonymous mutation (C > A) at codon 53 that leads to a premature Stop codon (TGC > TGA) and disrupts the juxtaposed cysteines (Cys53 – Cys54), typical of CC chemokines (Fig. 1a). Interestingly, in the Mexican, forest and Eastern cottontail rabbits, the Cys53 also presents a mutation, but it encodes a lysine (K). Despite this, all leporids studied present a frameshift mutation that disrupts exon 1 (Fig. 1a). In addition, Mexican cottontail presents a deletion of 20-base pairs (bp) at the beginning of exon 2 (Fig. 1b) that leads to another frameshift.

**Table 1:** Primers and conditions used for PCR amplification and sequencing of CCL16 from lagomorphs’ gDNA samples

	Species amplified	Primers sequence (5′- 3′)	Primer name	Exons amplified	PCR conditions	Fragment length
Leporids	Rabbits, hares and cottontails	CTCTCCCTGACACTGCTC	CCL16OrcuF1	1	95 °C (15 min) 40 cycles: 95 °C (45 s), 56 °C (15 s), 72 °C (10s) 60 °C (10 min)	~ 250 bp
		GCATAGTTCTGCTTGCAGA	CCL16OrcuR1.3			
	European rabbit, hares, cottontails, volcano rabbit	CAARGAGCRTGATTGACAG	CCL16Orcu F1d	2 + 3	95 °C (15 min) 40 cycles: 95 °C (45 s), 59 °C (20s), 72 °C (45 s) 60 °C (10 min)	~ 800 bp
		CCATTAGAAGGCCCAGCC	CCL16OrcuR1e			
	Riverine rabbit^b^	GTTCAGAGGCTGACGGCTC	CCL16OrcuF			
		CTGTGCAAATGCAGCCAGC	CCL16OrcuR1b			
	Amami rabbit^b^	GTTCAGAGGCTGACGGCTC	CCL16OrcuF		98 °C (3 min) 40 cycles: 98 °C (30s), 62 °C (20s), 72 °C (30s) 72 °C (5 min)	~ 893 bp
		CTTGTGGTCTGAGCCAGTGC	CCL16OrcuR1			
	Pygmy rabbit^b^	GTTCAGAGGCTGACGGCTC	CCL16OrcuF		95 °C (15 min) 40 cycles: 95 °C (45 s), 59 °C (20s), 72 °C (45 s) 60 °C (10 min)	
		CTTGTGGTCTGAGCCAGTGC	CCL16OrcuR1			
	All pikas	CATTCACAGTCCTCAGCCC	CCL16OcprF1^a^	1	95 °C (15 min) 40 cycles: 95 °C (45 s), 60 °C (20s), 72 °C (20s) 60 °C (10 min)	~ 230 bp
		GGTGGCAGAGAAGTGACAC	CCL16OcprR1			
	American pika, manchurian pika, turuchan pika	CATGTGTGAATCCAGAGGAG	CCL16OcprF2	2 + 3	95 °C (15 min) 40 cycles: 95 °C (45 s), 58 °C (20s), 72 °C (1 min) 60 °C (10 min)	~ 930 bp
		GAGGCAACACAATCACATTG	CCL16OcprR2^a^			
	Palla’s pika, Hoffmann’s pika, steppe pika, Northern pika	GTCAGCCGTCCTTGTTCACC	CCL16OcprF2a		95 °C (15 min) 40 cycles: 95 °C (45 s), 60 °C (20s), 72 °C (45 s) 60 °C (10 min)	~ 830 bp
		GTAAACCTGCACCAACATAGG	CCL16OcprR2a			

^a^ primers used for cDNA amplification; ^b^ As the PCRs were not successful for these species by using the same primers as for the European rabbit, new primers (CCL16F, CCL16R1 and CCL16R1b) were designed based on a consensus sequence obtained by comparing the European rabbit, hares, cottontails and volcano rabbit CCL16 sequences

Other species-specific mutations that can lead to pseudogenization were also observed. Indeed, there are some single nucleotide deletions for pygmy rabbit (position 147) and for the Amami rabbit (position 276), and all leporids present a single mutation at position 393 (Fig. 1a). Other deletions are also observed for all leporids (Fig. 1a). In exon 1 (signal peptide), frameshift deletions that disrupt the sequence are detected for the European, riverine and pygmy rabbits and hares (16 nucleotides), and for the volcano rabbit and cottontails (19 nucleotides). Furthermore, pygmy and riverine rabbits present other mutations that lead to premature stop codons. These mutations occur in the pygmy rabbit at nucleotide position 283 (GAG (Glu) > TAG) and in the riverine rabbit at position 319 (AGA (Arg) > TGA) (Fig. 1a). All these deletions were probably due to independent events that occurred at different moments in the evolution of leporids, and are likely to be lineage-specific. Amplification of *CCL16* from gDNA of the Amami rabbit also revealed an insertion of 24 nucleotides at the end of the second exon (from position 343 to 366) (Fig. 1a).

**Fig. 2:**
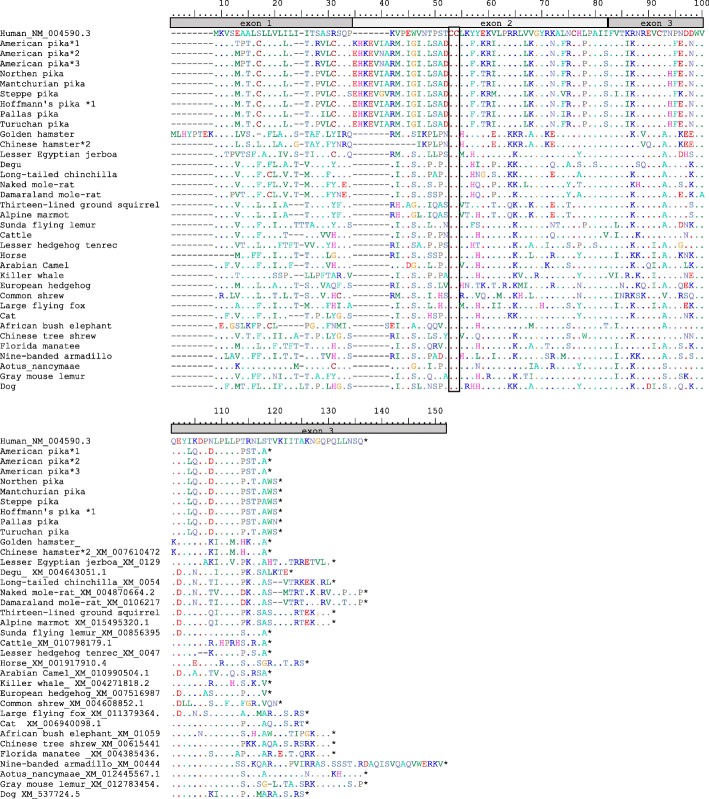
Amino acid alignment of CCL16 for several mammalian species. The characteristic Cys Cys motif is boxed. (*) represent normal Stop codons; (−) represent indels; *1, *2 and *3 represent different alleles. Human (*Homo sapiens*_NM_004590.3); Leporids: European rabbit (*Oryctolagus cuniculus cuniculus* _MK305138 and *O. c. algirus*_MK305139, MK305140*),* riverine rabbit (*Bunolagus monticularis*_MK305141), amami rabbit (*Pentalagus furnessi_*MK305136), pygmy rabbit (*Brachylagus idahoensis_*MK305131, MK305132*),* Mexican cottontail (*Sylvilagus cunicularis*_MK305145), forest cottontail (*S. brasiliensis*_MK305143, MK305144), Eastern cottontail (*S. floridanus*_MK305146, MK305147), European brown hare (*Lepus europaeus_*MK305133, MK305134*),* Iberian hare (*L. granatensis*_MK305135), volcano rabbit (*Romerolagus diazi*_MK305142); *Ochotona* species: American pika (*Ochotona princeps_*MK305156, MK305148, MK305149), Northern pika (*O. hyperborean*_MK305150), manchurian pika (*O. mantchurica_*MK305151), steppe pika (*O. pusilla_*MK305152), Hoffmann’s pika (*O. hoffmanni_* MK305155, MK305137), Palla’s pika (*O. pallasi_* MK305153), turuchan pika (*O. turuchanensis*_MK305154); Rodents: golden hamster (*Mesocricetus auratus*_XM_013118284.1), Chinese hamster (*Cricetulus griseus*_XM_007610472.2), lesser Egyptian jerboa (*Jaculus jaculus*_XM_012950139.1), Ord’s kangaroo rat (*Dipodomys ordii*_XM_013013071.1), guinea pig (*Cavia porcellus*_XM_005008470.1), degu (*Octodon degus*_XM_004643051.1), long tailed chinchilla (*Chinchilla lanigera*_XM_005415289.2), naked mole-rat (*Heterocephalus glaber*_XM_004870664.2), damara mole-rat (*Fukomys damarensis*_XM_010621757.1), thirteen-lined ground squirrel (*Ictidomys tridecemlineatus*_XM_005321496.2), sunda flying lemur (*Galeopterus variegatus*_XM_008563956.1); cattle (*Bos Taurus*_XM_010798179.1); lesser hedgehog tenrec (*Echinops telfairi*_XM_004707357.1); horse (*Equus caballus*_XM_001917910.4); Arabian camel (*Camelus dromedaries*_XM_010990504.1); killer whale (*Orcinus orca*_XM_004271818.2); European hedgehog (*Erinaceus europaeus*_XM_007516987.2); common shrew (*Sorex araneus*_XM004608852.1); large flying fox (*Pteropus vampyrus*_XM_011379364.1); cat (*Felis catus*_XM_006940098.1); African bush elephant (*Loxodonta Africana*_XM_010594587.1); Chinese tree shrew (*Tupaia belangeri chinensis*_XM_006154411.2); Florida manatee (*Trichechus manatus latirostris*_XM_004385436.1); nine-banded armadillo (*Dasypus novemcinctus*_XM_004449436.2); nancy Ma’s night monkey (*Aotus nancymaae*_XM_012445567.1); gray mouse lemur (*Microcebus murinus*_XM012783454.1); dog (*Canis lupus familiaris*_XM_537724.5). Numbering is according to human CCL16 sequence (GenBank accession number: NM_004590.3), with signal peptide and indels (indicated as (−)) being included in the numbering

**Fig. 3:**
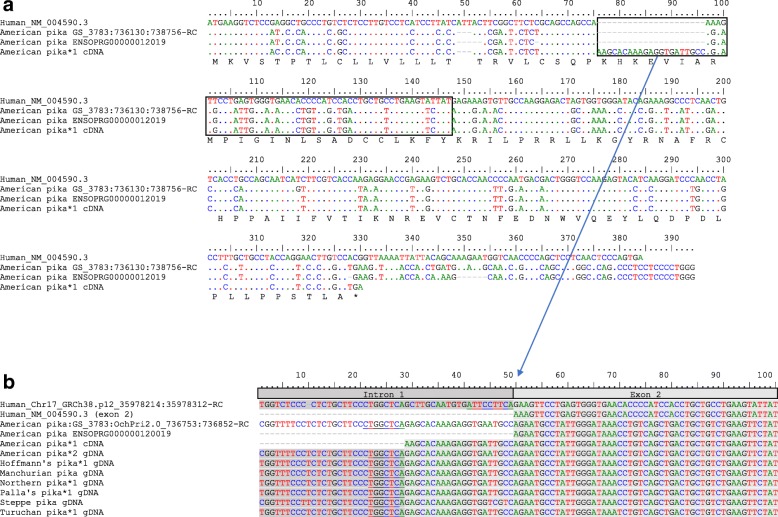
Comparison of the American pika CCL16 sequences retrieved from Ensembl (ENSOPRG00000012019) and obtained in this study (*1). The amino acid translation appears on the bottom. The beginning of exon 2 is boxed (**a**). Detail of the alternative splicing site in the American pika CCL16 gene, with predicted alternative splicing region underlined (**b**)

In the Ochotonidae family, with the exception of Hoffmann’s pika, *CCL16* seems to be functional (Fig. 2). In Hoffmann’s pika, *CCL16* encodes one functional allele, while the other presents the same mutation in the CC motif observed in leporids (Fig. 1a). Interestingly, some rodent species have a functional *CCL16* while in others it is pseudogenized, however due to mutations different than those described for lagomorphs. We successfully amplified the American pika *CCL16* from both cDNA and gDNA. The American pika sequence obtained from cDNA presented some differences when comparing with the sequence available in Ensembl (ENSOPRG00000012019; Fig. 3a). Indeed, it presents several indels and misses the stop codon, suggesting a non-functional *CCL16*. Our gDNA and cDNA sequences are in agreement with the American pika genomic sequence available in Gene Scaffold_3783:736130:738756:1, and seem to be functional, despite presenting an insertion of 21 nucleotides, that correspond to an insertion of seven amino acids, at the beginning of exon 2. The complete sequence of the *CCL16* gene (three exons and two introns) showed that this insertion derives from intron 1 (Fig. 3b) and might have resulted from the emergence of an alternative splicing site in the American pika *CCL16* gene. This alternative splicing site occurs in a CA motif located in the intron 21 bp upstream of the CA motif that immediately flanks the exon 2 of the human *CCL16* gene. These results were further confirmed by comparing the human and American pika *CCL16* gene sequences in NetGene2. Indeed, for the American pika, NetGene2 predicted that the splicing occurs at nucleotide position 28 while in human it corresponds to nucleotide position 49 (according to the human sequence; Fig. 3b). The remaining pikas also present an alternative splicing site at the same position as observed for the American pika.

In order to evaluate the evolutionary rates among Glires, we performed a Tajima relative rate test [29] where the CCL16 pseudogenes and non-pseudogenes (taxon B) were compared against other Glires with a functional CCL16. The *Homo sapiens* CCL16 sequence was used as outgroup. Our results (Table 2) show differences among these species, with pseudogenes presenting significantly higher number of nucleotide differences.

**Table 2:** Results obtained in Tajima Relative Rate Test using the human sequence as outgroup

Taxon A	Taxon B	Nr. differences in taxon A	Nr. differences in taxon B	Chi-square^1^	*p*-value^2^
Degu	Guinea pig	11	69	42.05	< 0.01
	Ord’s kangaroo rat	17	68	30.60	< 0.01
	European rabbit	16	72	35.64	< 0.01
	Long tailed chinchilla	18	10	2.29	0.13
	Lesser Egyptian jerboa	26	37	1.92	0.16
	Thirteen-lined ground squirrel	19	27	1.39	0.24

^1^Chi-square is a statistical test used to determine the substitution rates between species

^2^A p-value < 0.05 is used to reject the null hypothesis of equal rates between lineages

## Discussion

*CCL16* is a pseudogene in the European rabbit and in some rodents such as mouse, rat and guinea pig, but it seems to be functional in squirrel [20]. Our results showed that, as previously observed for the European rabbit, in the riverine rabbit, Amami rabbit, pygmy rabbit, European brown hare*,* Iberian hare, volcano rabbit, Mexican cottontail, Eastern cottontail and forest cottontail, *CCL16* is a pseudogene. We hypothesize that the Cys > Stop codon mutation appeared in the ancestor of leporids and reverted into a lysine in the cottontail branch at ~ 9.2 mya. Furthermore, there is a frameshift deletion at exon 1 in leporids that disrupts the sequence. Additionally, there are also other mutations originated from different pseudogenization events.

The complete cDNA sequence of the American pika *CCL16* gene showed an insertion derived from intron 1 that might have resulted from the emergence of an alternative splicing site. Human chemokines *CXCL12*, *CCL4*, *CCL20*, *CCL23* and *CCL27* also exhibit alternative splicing, leading to novel and functional proteins [30]. Alternative splicing is a crucial step in the mature mRNA production [31] and leads to protein diversity, being the major source of protein complexity in the immune system [31]. It occurs most frequently by exon skipping, mutually exclusive exons, alternative promoters or multiple polyadenylation sites, and alternative 5′ or 3′ spliced sites, and less frequently by intron retention [32, 33]. In the *Ochotona* spp*. CCL16* gene, alternative splicing seems to have occurred by intron retention, but its biological meaning remains to be determined.

For pikas, we observed that, with the exception of Hoffmann’s pika, the *CCL16* gene seems to encode a functional protein. Interestingly, in Hoffmann’s pika we identified two alleles, one corresponding to an intact gene and the other, similar to leporids, presenting a mutation in the Cys53 that leads to a premature stop codon. The similarity with the pseudogenization process observed in leporids suggests that this region may be prone to mutations. This is at odd as this site is important for disulfide bond formation, and thus alterations in this motif may alter protein structure and, consequently, its function.

CC chemokines are characterized by two juxtaposed cysteines that in CCL16 correspond to amino acids 53 and 54 of the mature protein (Fig. 2). The loss of one of these cysteines due to a mutation that encodes a premature stop codon leads to inactivation of this chemokine. Moreover, the mutation into an amino acid different than a Cys most likely impairs the protein to exert its functions. This is the case for all leporids studied and one allele of Hoffmann’s pika. The presence of the same mutation in the two families of the order Lagomorpha might be explained by parallel evolution in the different lineages such that the same mutation occurred independently at different time points in the lagomorphs’ evolution. Alternatively, this Cys - Stop mutation was already present in the lagomorphs’ ancestor and was later “distributed” stochastically, with some species presenting the stop mutation whilst others do not.

Considering that in rodents some species encode a functional CCL16 and in others *CCL16* is a pseudogene [20], we retrieved the available rodent CCL16 sequences from public databases (NCBI, Ensembl and UniProt). We observed that besides mouse, rat and guinea pig, *CCL16* might also be a pseudogene in the Ord’s kangaroo rat (Fig. 1). In these species, *CCL16* is a pseudogene due to different mutations. Mouse *CCL16* has been reported as a pseudogene due to mutations that lead to the loss of the characteristic juxtaposed conserved cysteines and an insertion of a Long Interspersed Element–1 (L1) in the third exon [23]. As for rat *CCL16* [20, 24], there is no further information on what led to its pseudogenization and no sequence is available in the public databases. For the Ord’s kangaroo rat and guinea pig, *CCL16* is a pseudogene due to premature stop codons at nucleotide positions 282 and 397, respectively. In the remaining available sequences, *CCL16* seems to encode a functional protein (Fig. 2).

**Fig. 4:**
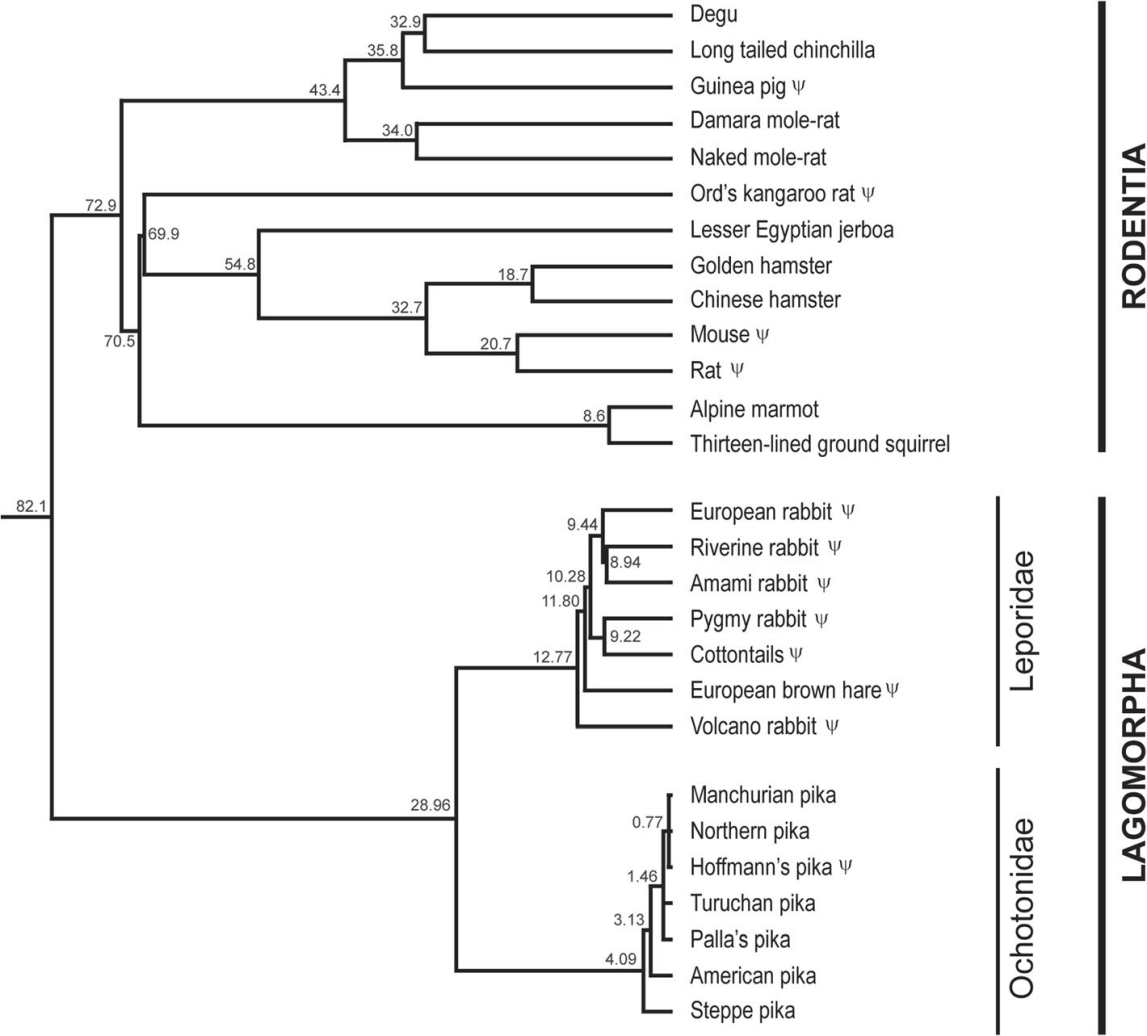
Phylogenetic relationships within the clade Glires. Divergence times (in million years ago) are indicated in the nodes and are according to [25, 27, 47]. Relationships within the Leporidae family are based on a molecular supermatrix (adapted from [27]), while for the Ochotonidae family it is based on a multilocus coalescent approach (adapted from [47]). Within Rodentia, relationships are according to [25]. ψ indicates the pseudogenes

The mutations observed in different rodents’ lineages may indicate that the *CCL16* gene was functional in the rodents’ ancestor and became later pseudogenized (Fig. 4). Indeed, we observed that Muridae (mouse and rat), Heteromyidae (Ord’s kangaroo rat) and Cavioidea (guinea pig) have a pseudogenized *CCL16* gene while in members of the Sciuroidea (thirteen-lined ground squirrel and alpine marmot), Cricetidae (Chinese and golden hamsters), Dipodidae (lesser Egyptian jerboa), Bathyergidae (naked mole-rat and damara mole-rat), Chinchilloidea (long tailed chinchilla), and Octodontoidea (degu), it is intact. Thus, the *CCL16* pseudogenization also occurred stochastically along the Rodentia order.

The Tajima relative rate test results rejected the null hypothesis, clearly showing that the *CCL16* pseudogenes are evolving faster than the non-pseudogenized *CCL16* genes, providing further evidence of an ongoing pseudogenization process in the Glires clade.

Usually, a gene is lost when it is removed from the genome or when it is still in the genome but with no functional role due to deleterious mutations (frameshift, deletions, insertions, early stop codons) [34]. The Black Queen Hypothesis [35] argues that the loss of a gene, although being deleterious, can be beneficial to the organism, mostly when related to pathogen resistance, being a pervasive process in all life kingdoms [34, 36]. Examples of this hypothesis are the resistance to acquired immunodeficiency syndrome (AIDS) and malaria in humans with mutations in the CCR5 and in the atypical chemokine receptor 1, respectively [34, 37]. In vertebrates, the number of chemokines varies among species [4], being characterized by the promiscuity of ligand-receptor binding and also by their chromosomal location and similar gene structure [38]. CCL16 is located in the MIP region of the CC cluster, which is important for immune cells recruitment [21], and is described in close vicinity of *CCL5*, *CCL14* and *CCL15* [20, 24]. Previous studies showed that, similar to *CCL16*, *CCL14* is a pseudogene for some lagomorphs while functional for others [22]. Interestingly, *CCL3*, that is also located in the MIP region and is a pseudogene in some species (human, rat, mouse, guinea pig), presents in the same region genes with similar structure and function, called *CCL3*-like genes [20]. This raises some hypotheses: *CCL16* loss in some rodents and leporids may be beneficial to these species (Black Queen hypothesis); the CCL16 functions’ may be replaced by other genes; some *CCL16*-like genes might be present in the genome. Additionally, we may speculate that the MIP region itself may be prone to gene loss events, being quite divergent among species [20].

## Conclusions

Overall these results suggest that in Glires (rodents and lagomorphs), *CCL16* suffered several independent pseudogenization events, with some species presenting one or both alleles disrupted. Thus, although *CCL16* was functional in the ancestor of the Glires clade, it became inactivated in some lineages. This may have occurred stochastically or in certain lineages at different times in the *CCL16* evolution, and could be associated with the CCL16 biological functions.

## Materials and methods

Genomic DNA was extracted according to the manufacturer’s instructions using the EasySpin Genomic DNA Minipreps Tissue Kit (Citomed, Torun, Poland) from tissue samples of European rabbit (*Oryctolagus cuniculus cuniculus and O. c. algirus),* riverine rabbit (*Bunolagus monticularis*), Amami rabbit (*Pentalagus furnessi*), pygmy rabbit (*Brachylagus idahoensis),* Mexican cottontail (*Sylvilagus cunicularis*), forest cottontail (*S. brasiliensis*), Eastern cottontail (*S. floridanus*), European brown hare (*Lepus europaeus),* Iberian hare (*L. granatensis*), volcano rabbit (*Romerolagus diazi*), American pika (*Ochotona princeps*), Northern pika (*O. hyperborea*), manchurian pika (*O. mantchurica*), steppe pika (*O. pusilla*), Hoffmann’s pika (*O. hoffmanni*), Palla’s pika (*O. pallasi*) and turuchan pika (*O. turuchanensis*). *Ochotona* samples were provided by Andrey A. Lissovsky, Zoological Museum of Moscow State University, Russia. The *Sylvilagus brasiliensis* sample was provided by Cibele Rodrigues Bonvicino, Instituto Nacional de Câncer (INCA), Brazil. The remaining samples were available in the CIBIO tissue samples collection. Approval from an ethics committee was unnecessary since no animals were killed for the purpose of this study and these samples have been described and used in previous publications [15, 19, 22, 39–41]. Total RNA was extracted from liver tissue of one specimen of American pika by using the RNeasy Mini Kit (Qiagen, Hilden, Germany) according to the manufacturer’s instructions. RNA quality, integrity and concentration (Table 3) were measured using NanoDrop. Further, RNA samples were ran into an agarose gel (0.8%). cDNA was synthesized according to the manufacturer’s instructions by using a total of 1 μg of RNA, oligo(dT) as primers and SuperScriptIII reverse transcriptase (Invitrogen, Carlsbad, CA, USA). The American pika (ENSOPRG00000012019) sequence available in Ensembl was used for primer design (Table 1), while for the European rabbit, we used the alignment of several mammalian *CCL16* sequences (Additional file 1 and Table 1). PCR amplification from gDNA was performed by amplification of several overlapping fragments with Multiplex PCR Kit (Qiagen, Hilden, Germany), according to the manufacturer’s protocol. Sequencing was performed on an ABI PRISM 310 Genetic Analyser (PE Applied Biosystems) and PCR products were sequenced in both directions*.* Sequences were submitted to GenBank under the following accession numbers: MK305131-MK305156.

**Table 3:** RNA samples concentrations

Sample ID	Nuclei Acid Concentration ng/uL	260/280
American pika	66.8	2.05

The sequences obtained were aligned with other CCL16 sequences available in GenBank. For Rodentia, all available sequences were used along with CCL16 sequences from the most representative mammalian orders (e.g. Primates, Artyodactyla, Carnivores, etc). Sequences were aligned using MUltiple Sequence Comparison by Log-Expectation (MUSCLE) available at http://www.ebi.ac.uk/ [42] and translated using BioEdit [43].

Splicing sites were predicted by using the NetGene2 server available at http://www.cbs.dtu.dk/services/NetGene2/ [44, 45].

The Tajima’s relative rate test [29] was conducted in MEGAX [46] in order to understand the evolutionary rate of Glires *CCL16* and its statistical significance. We used *CCL16* pseudogenes and non-pseudogenes as taxon B; for taxon A, species other than Degu (*Octodon degus*) were used. However, since similar results were obtained, only the results for Degu are presented.

## Additional file

Additional file 1: Alignment of the mammalian CCL16 sequences used for primer design for the leporids' PCR amplification. (PDF 25 kb)

## Abbreviations

AIDS: acquired immunodeficiency syndrome
bp: base pair
CCL16: C-C motif chemokine ligand 16
CCR: C-C motif chemokine receptor
cDNA: complementary DNA
DNA: Deoxyribonucleic acid
gDNA: genomic DNA
HCC: Human CC chemokine
LEC: Liver-expressed chemokine
MIP: Macrophage inflammatory protein
mRNA: Messenger RNA
MUSCLE: Multiple Sequence Comparison by Log-Expectation
Mya: Million years ago
PCR: Polymerase chain reaction
RNA: Ribonucleic acid

## References

[1] Ono SJ, Nakamura T, Miyazaki D, Ohbayashi M, Dawson M, Toda M (2003). Chemokines: roles in leukocyte development, trafficking, and effector function. J Allergy Clin Immunol.

[2] Zlotnik A, Yoshie O (2012). The chemokine superfamily revisited. Immunity.

[3] Nomiyama H, Hieshima K, Osada N, Kato-Unoki Y, Otsuka-Ono K, Takegawa S, Izawa T, Yoshizawa A, Kikuchi Y, Tanase S (2008). Extensive expansion and diversification of the chemokine gene family in zebrafish: identification of a novel chemokine subfamily CX. BMC Genomics.

[4] Nomiyama H, Osada N, Yoshie O (2013). Systematic classification of vertebrate chemokines based on conserved synteny and evolutionary history. Genes Cells.

[5] Crump MP, Spyracopoulos L, Lavigne P, Kim KS, Clark-lewis I, Sykes BD (1999). Backbone dynamics of the human CC chemokine eotaxin: fast motions, slow motions, and implications for receptor binding. Protein Sci.

[6] Teran LM (2000). CCL chemokines and asthma. Immunol Today.

[7] Van Coillie E, Van Damme J, Opdenakker G (1999). The MCP/eotaxin subfamily of CC chemokines. Cytokine Growth Factor Rev.

[8] Nomiyama H, Hieshima K, Nakayama T, Sakaguchi T, Fujisawa R, Tanase S, Nishiura H, Matsuno K, Takamori H, Tabira Y (2001). Human CC chemokine liver-expressed chemokine/CCL16 is a functional ligand for CCR1, CCR2 and CCR5, and constitutively expressed by hepatocytes. Int Immunol.

[9] Youn BS, Zhang S, Broxmeyer HE, Antol K, Fraser MJ, Hangoc G, Kwon BS (1998). Isolation and characterization of LMC, a novel lymphocyte and monocyte chemoattractant human CC chemokine, with myelosuppressive activity. Biochem Biophys Res Commun.

[10] Shields DC (2000). Gene conversion among chemokine receptors. Gene.

[11] Esteves PJ, Abrantes J, van der Loo W (2007). Extensive gene conversion between CCR2 and CCR5 in domestic cat (Felis catus). Int J Immunogenet.

[12] Vazquez-Salat N, Yuhki N, Beck T, O'Brien SJ, Murphy WJ (2007). Gene conversion between mammalian CCR2 and CCR5 chemokine receptor genes: a potential mechanism for receptor dimerization. Genomics.

[13] Perelygin AA, Zharkikh AA, Astakhova NM, Lear TL, Brinton MA (2008). Concerted evolution of vertebrate CCR2 and CCR5 genes and the origin of a recombinant equine CCR5/2 gene. J Hered.

[14] Carmo CR, Esteves PJ, Ferrand N, van der Loo W (2006). Genetic variation at chemokine receptor CCR5 in leporids: alteration at the 2nd extracellular domain by gene conversion with CCR2 in Oryctolagus, but not in Sylvilagus and Lepus species. Immunogenetics.

[15] Abrantes J, Carmo CR, Matthee CA, Yamada F, van der Loo W, Esteves PJ (2011). A shared unusual genetic change at the chemokine receptor type 5 between Oryctolagus, Bunolagus and Pentalagus. Conserv Genet.

[16] van der Loo W, Afonso S, de Matos AL, Abrantes J, Esteves PJ (2012). Pseudogenization of the MCP-2/CCL8 chemokine gene in European rabbit (genus Oryctolagus), but not in species of cottontail rabbit (Sylvilagus) and hare (Lepus). BMC Genet.

[17] van der Loo W, Magalhaes MJ, de Matos AL, Abrantes J, Yamada F, Esteves PJ (2016). Adaptive gene loss? Tracing Back the Pseudogenization of the rabbit CCL8 chemokine. J Mol Evol.

[18] de Matos AL, Lanning DK, Esteves PJ (2014). Genetic characterization of CCL3, CCL4 and CCL5 in leporid genera Oryctolagus, Sylvilagus and Lepus. Int J Immunogenet.

[19] Neves F, Abrantes J, Esteves PJ (2016). Evolution of CCL11: genetic characterization in lagomorphs and evidence of positive and purifying selection in mammals. Innate Immun.

[20] Shibata K, Nomiyama H, Yoshie O, Tanase S (2013). Genome diversification mechanism of rodent and Lagomorpha chemokine genes. Biomed Res Int.

[21] Zlotnik A, Yoshie O, Nomiyama H (2006). The chemokine and chemokine receptor superfamilies and their molecular evolution. Genome Biol.

[22] Neves F, Abrantes J, Lissovsky AA, Esteves PJ (2015). Pseudogenization of CCL14 in the Ochotonidae (pika) family. Innate Immun.

[23] Fukuda S, Hanano Y, Iio M, Miura R, Yoshie O, Nomiyama H (1999). Genomic organization of the genes for human and mouse CC chemokine LEC. DNA Cell Biol.

[24] Nomiyama H, Osada N, Yoshie O (2010). The evolution of mammalian chemokine genes. Cytokine Growth Factor Rev.

[25] Hedges SB, Marin J, Suleski M, Paymer M, Kumar S (2015). Tree of life reveals clock-like speciation and diversification. Mol Biol Evol.

[26] Blanga-Kanfi S, Miranda H, Penn O, Pupko T, DeBry RW, Huchon D (2009). Rodent phylogeny revised: analysis of six nuclear genes from all major rodent clades. BMC Evol Biol.

[27] Matthee CA, van Vuuren BJ, Bell D, Robinson TJ (2004). A molecular supermatrix of the rabbits and hares (Leporidae) allows for the identification of five intercontinental exchanges during the Miocene. Syst Biol.

[28] Lissovsky AA (2014). Taxonomic revision of pikas Ochotona (Lagomorpha, Mammalia) at the species level. Mammalia.

[29] Tajima F (1993). Simple methods for testing the molecular evolutionary clock hypothesis. Genetics.

[30] Colobran R, Pujol-Borrell R, Armengol MP, Juan M (2007). The chemokine network. II. On how polymorphisms and alternative splicing increase the number of molecular species and configure intricate patterns of disease susceptibility. Clin Exp Immunol.

[31] Shakola F, Suri P, Ruggiu M (2015). Splicing regulation of pro-inflammatory cytokines and chemokines: at the Interface of the neuroendocrine and immune systems. Biomolecules.

[32] Keren H, Lev-Maor G, Ast G (2010). Alternative splicing and evolution: diversification, exon definition and function. Nat Rev Genet.

[33] Sahoo A, Im SH (2010). Interleukin and interleukin receptor diversity: role of alternative splicing. Int Rev Immunol.

[34] Albalat R, Canestro C (2016). Evolution by gene loss. Nat Rev Genet.

[35] Morris JJ, Lenski RE, Zinser ER. The black queen hypothesis: evolution of dependencies through adaptive gene loss. MBio. 2012;3(2):e00036-12. 10.1128/mBio.00036-12. 10.1128/mBio.00036-12 PMC3315703 22448042

[36] Olson MV (1999). When less is more: gene loss as an engine of evolutionary change. Am J Hum Genet.

[37] Sharma V, Hecker N, Roscito JG, Foerster L, Langer BE, Hiller M (2018). A genomics approach reveals insights into the importance of gene losses for mammalian adaptations. Nat Commun.

[38] Wang J, Adelson DL, Yilmaz A, Sze SH, Jin Y, Zhu JJ (2005). Genomic organization, annotation, and ligand-receptor inferences of chicken chemokines and chemokine receptor genes based on comparative genomics. BMC Genomics.

[39] Abrantes J, Esteves PJ, Carmo CR, Muller A, Thompson G, van der Loo W (2008). Genetic characterization of the chemokine receptor CXCR4 gene in lagomorphs: comparison between the families Ochotonidae and Leporidae. Int J Immunogenet.

[40] van der Loo W, Abrantes J, Esteves PJ (2009). Sharing of endogenous lentiviral gene fragments among leporid lineages separated for more than 12 million years. J Virol.

[41] Surridge AK, van der Loo W, Abrantes J, Carneiro M, Hewitt GM, Esteves PJ (2008). Diversity and evolutionary history of the MHC DQA gene in leporids. Immunogenetics.

[42] Edgar RC (2004). MUSCLE: multiple sequence alignment with high accuracy and high throughput. Nucleic Acids Res.

[43] Hall TA (1999). BioEdit: a user-friendly biological sequence alignment editor and analysis program for Windows 95/98/NT. Nucl Acids Symp Ser.

[44] Brunak S, Engelbrecht J, Knudsen S (1991). Prediction of human mRNA donor and acceptor sites from the DNA sequence. J Mol Biol.

[45] Hebsgaard SM, Korning PG, Tolstrup N, Engelbrecht J, Rouze P, Brunak S (1996). Splice site prediction in Arabidopsis thaliana pre-mRNA by combining local and global sequence information. Nucleic Acids Res.

[46] Kumar S, Stecher G, Li M, Knyaz C, Tamura K (2018). MEGA X: molecular evolutionary genetics analysis across computing platforms. Mol Biol Evol.

[47] Melo-Ferreira J, Lemos de Matos A, Areal H, Lissovsky AA, Carneiro M, Esteves PJ (2015). The phylogeny of pikas (Ochotona) inferred from a multilocus coalescent approach. Mol Phylogenet Evol.

