# Supplementary material for: Evolution of CCL16 in Glires (Rodentia and Lagomorpha) shows an unusual random pseudogenization pattern
Source: BMC Evol Biol. 2019 Feb 20;19:59. doi: 10.1186/s12862-019-1390-7 (PMC6383237; doi:10.1186/s12862-019-1390-7)
Supplement: Supplementary file 1 — Alignment of the mammalian CCL16 sequences used for primer design for the leporids' PCR amplification. (PDF 25 kb) [file 12862_2019_1390_MOESM1_ESM.pdf]

Supplementary material 1.

```
                                                            10        20        30        40        50        60        70        80        90       100
                                                   ....|....|....|....|....|....|....|....|....|....|....|....|....|....|....|....|....|....|....|....|
Rabbit_OryCun2.0_NC_013687.1(24956224-24959622)-RC TCTGCACCAGGCCCCATCCTGTGCCACCCTCCCTCTCAGCCAACCCTTGAAGCGGCCACAGTCCACAGCTGGAGGGCAGCTCAGCAGAGCCCTGCTCCGC
Human_NM_004590.3                                  ----------------------------------------------------------------------------------------------------
American Pika_ENSOPRT00000012014.1                 ----------------------------------------------------------------------------------------------------
Golden hamster_XM_013118284.2                      ----------------------------------------------------------------------------------------------------
Gray mouse lemur_XM_012783454.2                    ----------------------------------------------------------------------------------------------------
Rhesus macaque_XM_001112513.3                      ----------------------------------------------------------------------------------------------------
Cattle_XM_024980640.1                              ----------------------------------------------------------------------------------------------------
Lesser hedgehog tenrec_XM_004707357.1              ----------------------------------------------------------------------------------------------------
Killer whale_ XM_004271818.2                       ----------------------------------------------------------------------------------------------------
Large flying fox_XM_011379364.1                    ----------------------------------------------------------------------------------------------------
Florida manatee _XM_004385436.1                    ----------------------------------------------------------------------------------------------------
Aotus_nancymaae_XM_012445567.1                     ----------------------------------------------------------------------------------------------------

                                                           110       120       130       140       150       160       170       180       190       200
                                                   ....|....|....|....|....|....|....|....|....|....|....|....|....|....|....|....|....|....|....|....|
                                                           [                                        exon 1                                            ]
Rabbit_OryCun2.0_NC_013687.1(24956224-24959622)-RC TCTCCCTGACACTGCTCTGGCCCTCCAGGAGAATGAAGGTCTCCGTGGCTGCCCTCTGCCTCCTCGTCCTCCTCCT----------------TTTTGCAG
Human_NM_004590.3                                  --------------------------------.............A.........G.CT.....T......A....TATCATTACTTCGGCT.C.C....
American Pika_ENSOPRT00000012014.1                 --------------------------------............A..C.CA.............T...........------CACCTCGAGT.C.C....
Golden hamster_XM_013118284.2                      --------.TG....A..AC...A..GA..AG............T...TCT.....CT.T......C.A.....AGCACAGCCTTCGCTCTC.ACATTC.
Gray mouse lemur_XM_012783454.2                    --------------------------------...........T.........T...TT......AA.A..A....CACCACTACTGCTGCT....A...
Rhesus macaque_XM_001112513.3                      --------------------------------.............A.........G.CT......A.....A....TATCATTACTTCTGCT.C......
Cattle_XM_024980640.1                              --------------------------------.........................TT........T...AC.A.------CACTTCTGTTG..CA...
Lesser hedgehog tenrec_ XM_004707357.1             --------------------------------...............A........CT..........T..ACAT.CACTGTGGTCTCTGCT.A.CA...
Killer whale_ XM_004271818.2                       --------------------------------............AC...........CT......AG.TCA.CAT.A---CTTCTGCCATTCACAGC...
Large flying fox_XM_011379364.1                    --------------------------------...........T.CA..........TT......A.....A....CACCATGACTTCTGCT...CA..T
Florida manatee _XM_004385436.1                    --------------------------------............A...........CT.......T.....ACAT.CACGACTACTTCTGCT.C.CA...
Aotus_nancymaae_XM_012445567.1                     --------------------------------............AA.........G.CT............A....CATGATTACTTCTGCT.C......

                                                          210       220       230       240       250       260       270       280       290       300
                                                   ....|....|....|....|....|....|....|....|....|....|....|....|....|....|....|....|....|....|....|....|
                                                   [exon 1]
Rabbit_OryCun2.0_NC_013687.1(24956224-24959622)-RC CCAGCCAAGTGAGCCGGCTTGTCCCTGCAGGGCAGGCCTCAGAAAGCAGAATGGGCAGGTGGCAGAGAGCTGACACCAGGAAGGCTTTCAGGTTCTGGCC
Human_NM_004590.3                                  .......---------------------------------------------------------------------------------------------
American Pika_ENSOPRT00000012014.1                 .......---------------------------------------------------------------------------------------------
Golden hamster_XM_013118284.2                      ....AG.---------------------------------------------------------------------------------------------
Gray mouse lemur_XM_012783454.2                    .......---------------------------------------------------------------------------------------------
Rhesus macaque_XM_001112513.3                      .......---------------------------------------------------------------------------------------------
Cattle_XM_024980640.1                              .......---------------------------------------------------------------------------------------------
Lesser hedgehog tenrec_ XM_004707357.1             .......---------------------------------------------------------------------------------------------
Killer whale_ XM_004271818.2                       G..AGTG---------------------------------------------------------------------------------------------
Large flying fox_XM_011379364.1                    ...AG..---------------------------------------------------------------------------------------------
Florida manatee _XM_004385436.1                    ....T..---------------------------------------------------------------------------------------------
Aotus_nancymaae_XM_012445567.1                     .......---------------------------------------------------------------------------------------------
```

```
                                                                    2010      2020      2030      2040      2050      2060      2070      2080      2090      2100
                                                           ....|....|....|....|....|....|....|....|....|....|....|....|....|....|....|....|....|....|....|....|
                                                                                                                                          [ exon 2 ]
Rabbit_OryCun2.0_NC_013687.1(24956224-24959622)-RC         GGAGCGTGATTGACAGGAGATGGAGGTAGGCAGCCATGGTCTGCCTCCCTGCTCCCCATCATCCAATGTGATGATTGCAAAAATTCCTGACGGGGTAGAC
Human_NM_004590.3                                          -------------------------------------------------------------------------------...G.......GT....GA..
American Pika_ENSOPRT00000012014.1                         -------------------------------------------------------------------------------.G...G...ATT...A..A..
Golden hamster_XM_013118284.2                              ----------------------------------------------------------------------------------..G.....GTC.A..A.A
Gray mouse lemur_XM_012783454.2                            -------------------------------------------------------------------------------...........GTCA..GA..
Rhesus macaque_XM_001112513.3                              -------------------------------------------------------------------------------...G.......GT....GA..
Cattle_XM_024980640.1                                      -------------------------------------------------------------------------------...........GTC...GA..
Lesser hedgehog tenrec_XM_004707357.1                      -------------------------------------------------------------------------------.G......G..GTC..CCA..
Killer whale_ XM_004271818.2                               -------------------------------------------------------------------------------...........GTC...GA..
Large flying fox_XM_011379364.1                            -------------------------------------------------------------------------------...........GTC...GA.T
Florida manatee _XM_004385436.1                            -------------------------------------------------------------------------------...........GTCA..GA..
Aotus_nancymaae_XM_012445567.1                             -------------------------------------------------------------------------------...G.......G.....GA..

                                                                    2110      2120      2130      2140      2150      2160      2170      2180      2190      2200
                                                           ....|....|....|....|....|....|....|....|....|....|....|....|....|....|....|....|....|....|....|....|
                                                           [                                          exon 2                                                  ]
Rabbit_OryCun2.0_NC_013687.1(24956224-24959622)-RC         TATCCACCCCACTGATGCCTGAAGTTCCATGAGAAAGCATTGCCGGGGAAACTGGTGCCAGGATCCAGAAGGGCCCTCAGCTGCCACTTGCCAGTGGTCA
Human_NM_004590.3                                          ACC...T..AC...C..........ATT.........TG.....AA...G...A...GTG....A.....A........A...T...C......CAA...
American Pika_ENSOPRT00000012014.1                         CTGT..G.TG....C..T.........T..A...G.AT.C....AA...G....C..AA...C.A.C.G.AT..AT....A......CCA....CAA...
Golden hamster_XM_013118284.2                              CC..T....A....T....AT....A.T.........A.C.....AA.....G....G.G....A..A.GA........A.......C......CAA...
Gray mouse lemur_XM_012783454.2                            C.A...T..GC...C..........AT.......G..TG.....AA........ACTAT.....A.C...A........A...T...C......CAA...
Rhesus macaque_XM_001112513.3                              ACC...T..AC...C...........TT.........TG.....AA...G...A...GTG....A.....A........A...TA..C......CAA...
Cattle_XM_024980640.1                                      CCC......A....C..........AT..........T.......A...........GTG....A....CA........A...T...C....G.CAA..G
Lesser hedgehog tenrec_XM_004707357.1                      CCC......TC...C.........CA.ACG.......TG.....CA...........G.G....A..T..A............T.C.C.....TCAA...
Killer whale_ XM_004271818.2                               CC.......GC...C..........AT..........T......AA.A...G.....GTGA...A.....A........A....T..C......CAA...
Large flying fox_XM_011379364.1                            CT.....A.AC...C...A......AT.........AT.A.A..AA...........GTG...CA.....A............TT..C......CAA...
Florida manatee _XM_004385436.1                            C.A.G.GT.AC...C..........AT........G.T......CA...........G......A..CG.A............T...C......CAA...
Aotus_nancymaae_XM_012445567.1                             ATC......AC...C..........ATA.........TG.....AA...G.......ATT.CG.A......A..T....A...TT..C......CAA...

                                                                    2210      2220      2230      2240      2250      2260      2270      2280      2290      2300
                                                           ....|....|....|....|....|....|....|....|....|....|....|....|....|....|....|....|....|....|....|....|
                                                           []
Rabbit_OryCun2.0_NC_013687.1(24956224-24959622)-RC         TATGAGCACTTGCAGGTGTGTGTAAGAGGAAAGGGGAGGCTGGGGCAGGAACAGGGGGAGTGGGACCAAGACGTCTCCACTCTAGAGCTATGGAGTAGCG
Human_NM_004590.3                                          .C--------------------------------------------------------------------------------------------------
American Pika_ENSOPRT00000012014.1                         .T--------------------------------------------------------------------------------------------------
Golden hamster_XM_013118284.2                              .C--------------------------------------------------------------------------------------------------
Gray mouse lemur_XM_012783454.                             .C--------------------------------------------------------------------------------------------------
Rhesus macaque_XM_001112513.3                              .C--------------------------------------------------------------------------------------------------
Cattle_XM_024980640.1                                      .C--------------------------------------------------------------------------------------------------
Lesser hedgehog tenrec_XM_004707357.1                      .C--------------------------------------------------------------------------------------------------
Killer whale_ XM_004271818.2                               .C--------------------------------------------------------------------------------------------------
Large flying fox_XM_011379364.1                            .C--------------------------------------------------------------------------------------------------
Florida manatee _XM_004385436.1                            .C--------------------------------------------------------------------------------------------------
Aotus_nancymaae_XM_012445567.1                             .C--------------------------------------------------------------------------------------------------
```

```
                                                              2410      2420      2430      2440      2450      2460      2470      2480      2490      2500
                                                     ....|....|....|....|....|....|....|....|....|....|....|....|....|....|....|....|....|....|....|....|
                                                                                                                        exon 3
Rabbit_OryCun2.0_NC_013687.1(24956224-24959622)-RC   GAGGTGGGAGCCGTGGCCTAGACCTCAGCCTACTCAGCAGCAAGACCACAAACTCCGCCTCCACAGTTTGTCAACATAAAAA-CCTGGAGGTCTGCACCA
Human_NM_004590.3                                    ------------------------------------------------------------------..C....C..AG.GG.A..GA..A..........
American Pika_ENSOPRT00000012014.1                   ------------------------------------------------------------------..C....C......G.A..GT.............
Golden hamster_XM_013118284.2                        ------------------------------------------------------------------.......C..AG..G.ATG.A..A.....TG...
Gray mouse lemur_XM_012783454.2                      ------------------------------------------------------------------..CA...C..A...G.A..GA..A.......G..
Rhesus macaque_XM_001112513.3                        ------------------------------------------------------------------..CA...C..A..GG.A..AA..A..........
Cattle_XM_024980640.1                                ------------------------------------------------------------------..CA...C..AGCGG.AA.GA........T....
Lesser hedgehog tenrec_XM_004707357.1                ------------------------------------------------------------------..C....C..A...G.A..GA...A.....G.T.
Killer whale_ XM_004271818.2                         ------------------------------------------------------------------G.CA...C..G..GG.AA.GA...A....T....
Large flying fox_XM_011379364.1                      ------------------------------------------------------------------..CA...C..A...G.AT.GA...A.....G...
Florida manatee _XM_004385436.1                      ------------------------------------------------------------------..CA...C..A..GG.A..GA...A....TG...
Aotus_nancymaae_XM_012445567.1                       ------------------------------------------------------------------..CA...CA.A...G.A..GA..A.......G..

                                                              2510      2520      2530      2540      2550      2560      2570      2580      2590      2600
                                                     ....|....|....|....|....|....|....|....|....|....|....|....|....|....|....|....|....|....|....|....|
                                                                                                     exon 3
Rabbit_OryCun2.0_NC_013687.1(24956224-24959622)-RC   ACTCTGAAGACACCCGGGCCCAAGAGCACCTCCAGGATCCCTGGCCACCTCTGCTGCCTGCCAGAACCCCAGCCCTGGTTAAAACCATCGGATCAGAGAG
Human_NM_004590.3                                    ..C.CA.T...GA.T...T.......T..A..A........AAC.T....T........A....G.A.TTGT..AC........TT..TAC.G..A...A
American Pika_ENSOPRT00000012014.1                   ...TC.......A.T...T.......T.............TGAC.TG......T.....C....T....TG...GAA............AA.AGCA.CCA
Golden hamster_XM_013118284.2                        ..C..A.G..GGAAT...T.A....AT..A..A........AAAAT....T..A.....CA..AG.ATTT....TA.
Gray mouse lemur_XM_012783454.2                      ..C.CAGCA.AGA.T...T.......T.TA..A........AAC.T....T.......CT......A.T.G.GG---C.......TGCAAC....AG..A
Rhesus macaque_XM_001112513.3                        ..C.CAGT...GA.T...T.......T..A..A..A.....AAC.T....T.......CT....G.A.TTG...AC........TT..TAC.G..A...A
Cattle_XM_024980640.1                                ..C.CA.CA.TGA.T...T.......T..A..A........A.A.TT.A..C..G..A.T....G.GGTTG...TA.
Lesser hedgehog tenrec_XM_004707357.1                ..C.C.GT...GATT...T.......T..A..A..------AA..T............CC....G.G..TG...TGA
Killer whale_ XM_004271818.2                         ..C.CA.TA..GAAT...T......AT..A..A........A.A.T....T...AT...T....G.AGTTG.T.TA.
Large flying fox_XM_011379364.1                      .TC.CA.T..A.AAT...T......TT..A..A.T...T..AAT.TG...T....A..C.....G.A.ATG....G.......T....TA.....T..
Florida manatee _XM_004385436.1                      ..C.CAGT....AAT...T...G...T..A..A........A.C.T....T.....G.CC....G.A.TTG...AG....G...TT.CTAC.CA.AG..A
Aotus_nancymaae_XM_012445567.1                       ..C.CA.C..TGA.T...T.......T..A..A........AAC.TG...T.......CT....G.A.TTG...AC........TA..TAC.G..A.C.A

                                                            2610      2620      2630      2640      2650      2660      2670      2680      2690      2700
                                                     ....|....|....|....|....|....|....|....|....|....|....|....|....|....|....|....|....|....|....|....|
                                                                  exon 3
Rabbit_OryCun2.0_NC_013687.1(24956224-24959622)-RC   GGGACAGCCCCAGCCCCAGCCCCTCTTCCATTAGAAGGCCCAGCCTAGGTAGGAGCCGGGGTTTGGGGAGGAGGGGTAGCCCTGTGCAAATGCAGCCAGC
Human_NM_004590.3                                    T..T..A.......T..TCAA.TC.CAGTGA
American Pika_ENSOPRT00000012014.1                   .CCC......TG...AGC...TC..C..C.GG.
Golden hamster_XM_013118284.2
Gray mouse lemur_XM_012783454.2                      A..C..A.......T..TAAG.TC.CCATGA
Rhesus macaque_XM_001112513.3                        T.ACT.A
Cattle_XM_024980640.1
Lesser hedgehog tenrec_XM_004707357.1
Killer whale_ XM_004271818.2
Large flying fox_XM_011379364.1
Florida manatee _XM_004385436.1                      A..T..A...T..
Aotus_nancymaae_XM_012445567.1                       T..C..A...A...A..TCAA.TC.CAGT.A
```
